# Chromatin organization regulates viral egress dynamics

**DOI:** 10.1038/s41598-017-03630-y

**Published:** 2017-06-16

**Authors:** Vesa Aho, Markko Myllys, Visa Ruokolainen, Satu Hakanen, Elina Mäntylä, Jori Virtanen, Veijo Hukkanen, Thomas Kühn, Jussi Timonen, Keijo Mattila, Carolyn A. Larabell, Maija Vihinen-Ranta

**Affiliations:** 1 Department of Physics, and Nanoscience Center, University of Jyväskylä, Jyväskylä, Finland; 2 Department of Biological and Environmental Science, and Nanoscience Center, University of Jyväskylä, Jyväskylä, Finland; 3 Institute of Biotechnology, University of Helsinki, Helsinki, Finland; 4 Faculty of Medicine, University of Turku, Turku, Finland; 5 Department of Applied Physics, University of Eastern Finland, Kuopio, Finland; 6 Finnish Meteorological Institute, Kuopio, Finland; 7 ITMO University, Saint Petersburg, Russia; 8 Department of Physics, Tampere University of Technology, Tampere, Finland; 9 Department of Anatomy, University of California San Francisco, San Francisco, California USA; 10 Physical Biosciences Division, Lawrence Berkeley National Laboratory, Berkeley, California USA

## Abstract

Various types of DNA viruses are known to elicit the formation of a large nuclear viral replication compartment and marginalization of the cell chromatin. We used three-dimensional soft x-ray tomography, confocal and electron microscopy, combined with numerical modelling of capsid diffusion to analyse the molecular organization of chromatin in herpes simplex virus 1 infection and its effect on the transport of progeny viral capsids to the nuclear envelope. Our data showed that the formation of the viral replication compartment at late infection resulted in the enrichment of heterochromatin in the nuclear periphery accompanied by the compaction of chromatin. Random walk modelling of herpes simplex virus 1–sized particles in a three-dimensional soft x-ray tomography reconstruction of an infected cell nucleus demonstrated that the peripheral, compacted chromatin restricts viral capsid diffusion, but due to interchromatin channels capsids are able to reach the nuclear envelope, the site of their nuclear egress.

## Introduction

DNA viruses target the nucleus due to their dependence on the cellular DNA reproduction machinery, and the viral infection induces profound modifications of nuclear structures including chromatin. In lytic herpes simplex virus 1 (HSV-1) infection, the injection of the viral DNA into the nucleoplasm is followed by the formation of several small viral replication compartments (VRCs)^1, 2^. Later in infection, viral DNA replication and the accumulation of viral proteins is accompanied by the fusion of small VRCs into an enlarged VRC and increase in the nuclear volume^3, 4^. At the same time, with the emergence of the extensive VRC, the host chromatin is relocated into the nuclear periphery^5–7^. Chromatin marginalization that correlates with the expansion of the VRC is also seen in parvovirus-^8^ and baculovirus-^9^ infected cells. The progress of HSV-1 infection and the marginalization of host chromatin are accompanied by changes in the host gene expression^10^. Earlier studies have revealed that, instead of an HSV-1-induced general shut-down of cellular genes, the expression of some genes is maintained, or even increased in infection^10–16^. The final nuclear steps of infection are the assembly of viral capsids and their egress by budding through the inner nuclear membrane into the perinuclear space, followed by subsequent fusion with the outer nuclear membrane^17–20^. To reach the inner nuclear membrane, viral capsids have to penetrate through the layer of marginalized host chromatin.

Despite many achievements in the research of virus-nucleus interactions, prior studies have not provided sufficient details on the spatial and molecular organization of chromatin to elucidate whether chromatin constitutes an accessibility barrier for the translocation of viral capsids towards the inner nuclear membrane. Thus, we investigated the detailed structural organization of chromatin at late infection. First, to gain insight into the spatial localization of chromatin and viral capsids, we used confocal and transmission electron microscopy (TEM) imaging. Next, we analysed the molecular organization of chromatin using soft x-ray tomography (SXT), which allows for assessment of the composition and structure of chromatin by providing a quantitative, linear measure of its density^21, 22^. Finally, we created high-resolution 3D SXT reconstructions of the chromatin of infected cell nuclei and used them for numerical modelling of the viral capsid-sized particle mobility in the chromatin using a random walk model. These studies revealed that the spatial organization and density of chromatin change in the late stage of infection and that these structural changes restrict the mobility of capsids. This demonstrates that low-density channels through the chromatin are needed to allow for the passage of progeny capsids to the nuclear envelope (NE).

## Results

### Marginalization of chromatin at late infection

**Figure 1:**
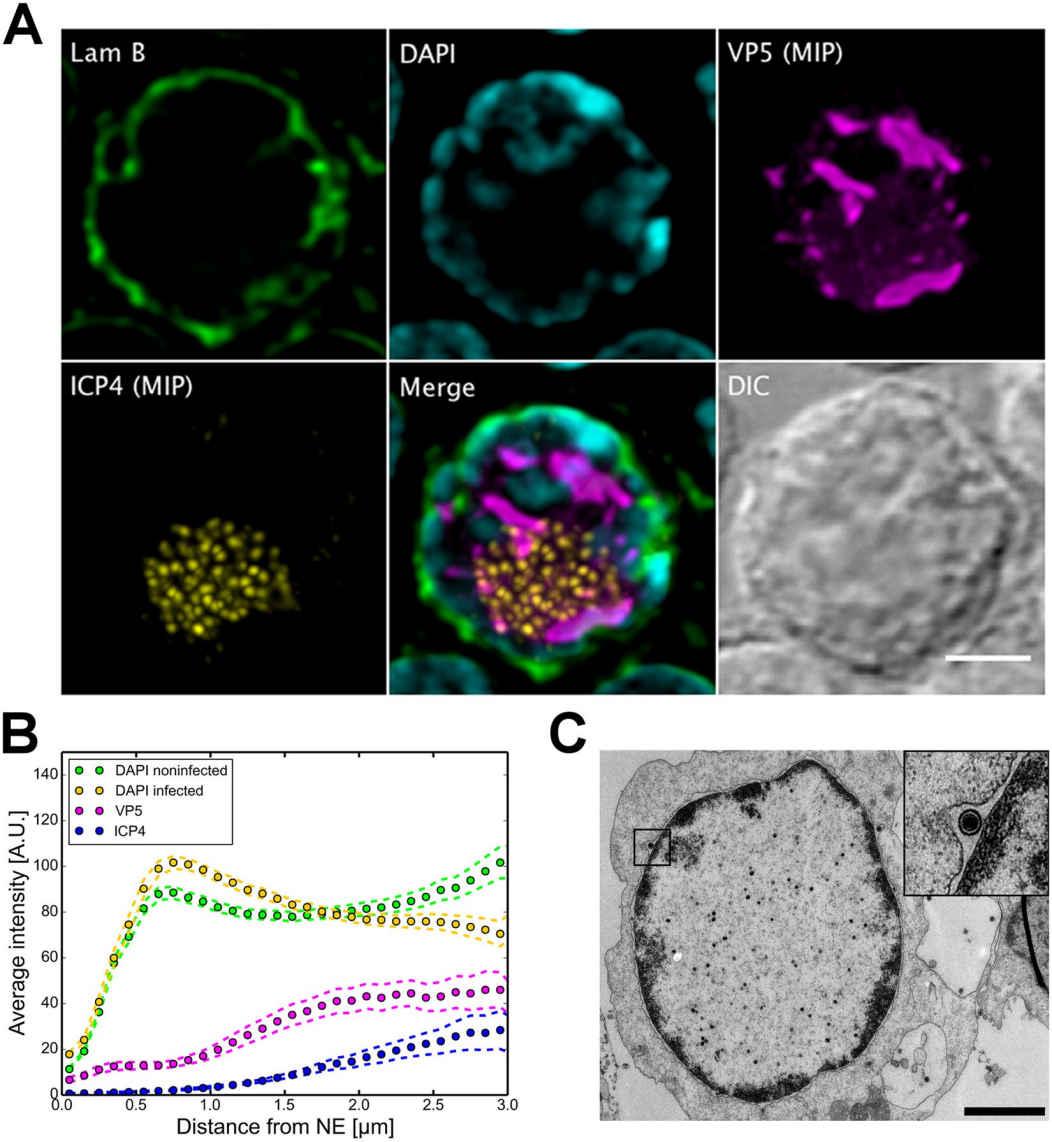
Nuclear distribution of host chromatin in infected cells with enlarged VRCs. (**A**) Confocal microscopy images showing the distribution of DAPI-labelled chromatin (cyan) and lamin B (green) together with maximum intensity projections (MIP) of viral EYFP-ICP4 (yellow) and VP5 (magenta) proteins at 24 h p.i. A differential interference contrast (DIC) image is also shown. Scale bar, 3 µm. (**B**) The mean spatial distribution of chromatin in infected (n = 16) and non-infected (n = 20) cells together with VP5 and ICP4 shown as plots of the intensity at increasing distances from the NE. Dotted error lines represent the mean ± the standard error of the mean (SEM). (**C**) A TEM image of an infected cell nucleus at 24 h p.i. The inset shows an enlarged view of the boxed area containing the viral capsid in the space between the inner and outer leaflets of the nuclear membrane. Scale bar, 3 µm.

The analysis of immunolabelled cells showed that at 24 h post infection (p.i.), 4′,6-diamidino-2-phenylindole (DAPI)-labelled chromatin was concentrated at the nuclear periphery. At the same time, the viral immediate early protein EYFP-ICP4 accumulated into distinct small foci inside the VRC, whereas the capsid protein VP5 was located throughout the VRC. In contrast to control cells (Supplementary Figure S1), lamin B staining appeared discontinuous along the nuclear rim (Fig. 1A). The total volumes occupied by the viral proteins in the inner regions of the nucleus were 15 ± 10 µm^3^ and 42 ± 20 µm^3^ (n = 20) for ICP4 and VP5, respectively, while 0.24% of ICP4 and 8.8% of VP5 were located in the nuclear periphery (the region between the DAPI label and NE). In some cells, EYFP-ICP4 and VP5 were also found to concentrate to the nuclear periphery (Supplementary Figure S2). Quantitative analysis of confocal microscopy data as a function of increasing distance from the NE confirmed the accumulation of chromatin to the region nearest to the NE in the infected cells. The amount of chromatin decreased towards the nuclear centre in infected cells, whereas in the non-infected cells chromatin was distributed more evenly throughout the nucleus (Fig. 1B). Here, although the VRC is in general devoid of the DNA-binding fluorochrome DAPI^23^, we cannot rule out the possibility that a small portion of the host chromatin might be located in the same area with the VRC. In all the infected nuclei examined (n = 16), the majority of ICP4 and VP5 were located in the nuclear centre, with only a small fraction of the proteins located in a 1 µm-thick chromatin region next to the lamin B layer and to the NE.

Consistent with the confocal data, TEM analysis demonstrated that at 24 h p.i. the host chromatin was marginalized in close proximity to the NE. Viral capsids were typically located in the centre of the nucleus in the enlarged VRC area, and a portion of capsids was found in the gaps between the two nuclear membranes (Fig. 1C). In addition, our earlier studies indicated the presence of capsids in the virus-induced low-density chromatin breakages penetrating the peripheral chromatin^7^. In summary, confocal analysis combined with TEM confirmed that the emergence of an enlarged VRC was accompanied by the accumulation of chromatin at the nuclear periphery.

### Distribution of modified histones

**Figure 2:**
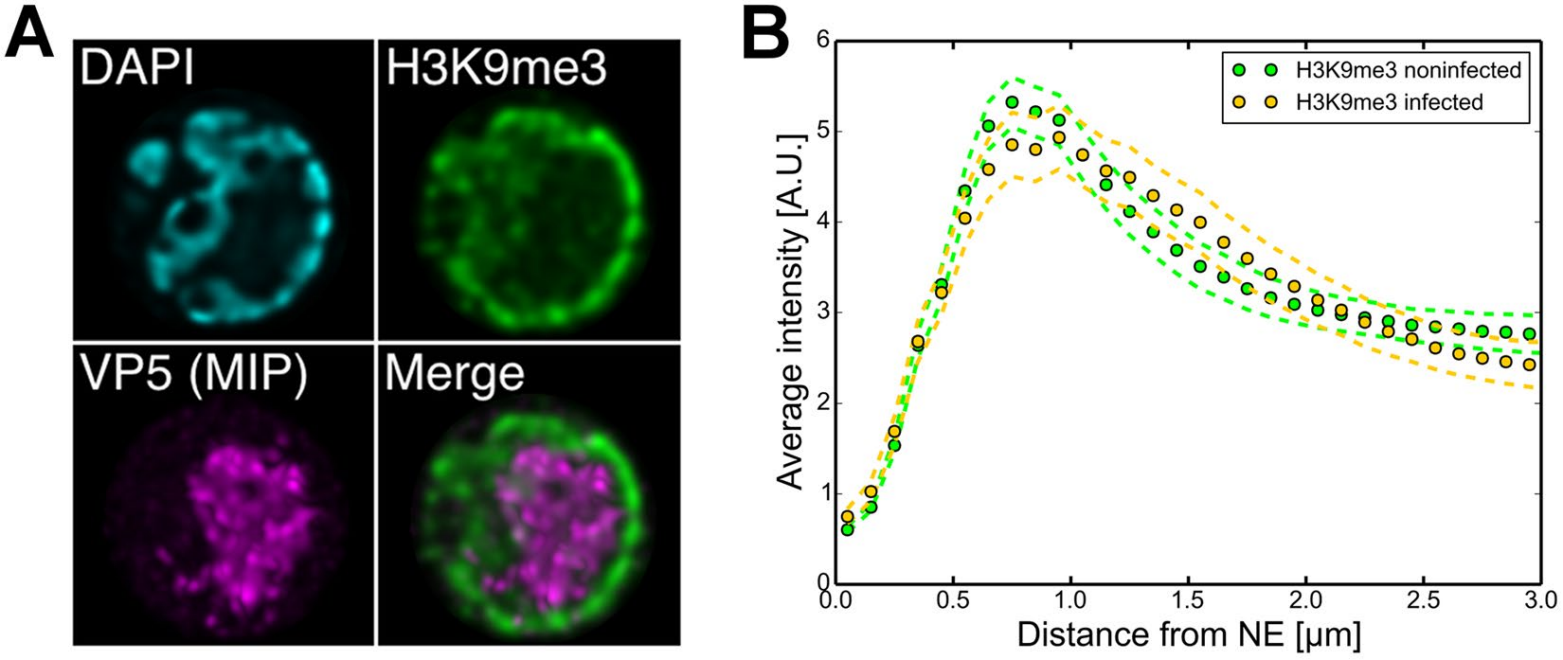
Redistribution of heterochromatin marker. (**A**) Confocal microscopy images of an infected cell with an enlarged VRC, stained with antibodies for VP5 (magenta), heterochromatin marker H3K9me3 (green) and DAPI (cyan) at 24 h p.i. See also Supplementary Figure S3. (**B**) Plot of the intensity of H3K9me3 in infected (yellow) and non-infected (green) cells as a function of the distance from the NE. Dotted error lines represent the mean ± SEM.

Histone proteins assemble DNA into nucleosomes, whose composition and spacing contribute to chromatin packing of higher order. We used histone H3 trimethyl Lys9 (H3K9me3) as a marker of transcriptional silencing to identify the tightly packed heterochromatin^24, 25^. Immunolabelling showed that this marker was located near the nuclear periphery in both the infected cells at late infection and in the non-infected cells (Fig. 2A, Supplementary Figure S3). We quantified the distribution of the chromatin marker by plotting fluorescence intensity as a function of distance from the NE detected with DAPI (Fig. 2B). The analysis showed that in both the infected and non-infected cells the H3K9me3 signal was strongest at the nuclear periphery and the infection did not cause major changes in the distribution of heterochromatin in comparison with that of non-infected cells. This suggested that the observed shift in the DAPI from the central regions of infected nuclei towards the nuclear periphery (Fig. 1B) was mainly caused by the exclusion of euchromatin to the periphery due to the emergence of an enlarged VRC.

### Virus-induced compaction and reorganization of the host chromatin

To further study the architecture of the nucleus, we used SXT to image hydrated cells in the near-native state. SXT image contrast is based on the absorption of x-rays by mainly carbon and nitrogen. This allows the measurement of the linear absorption coefficient (LAC) of cell structures, which reflects the concentration of cellular biomolecules^26, 27^. Because of the increased density of biomolecules in heterochromatin, the heterochromatin region of the nucleus has a higher LAC than the less densely packed euchromatin, as is evident from computer-generated SXT orthoslices through nuclei (Supplementary Movie S1). The EYFP-ICP4 HSV-1 strain allowed detection of the VRC and identification of the infected cells by cryogenic fluorescence microscopy (CFM)^7^. When SXT orthoslices were aligned with CFM images of the same cell^28^, EYFP-ICP4 was found to be localized in distinct nuclear foci or in a few enlarged foci in the heterochromatin-depleted regions of the nuclei^7^.

**Figure 3:**
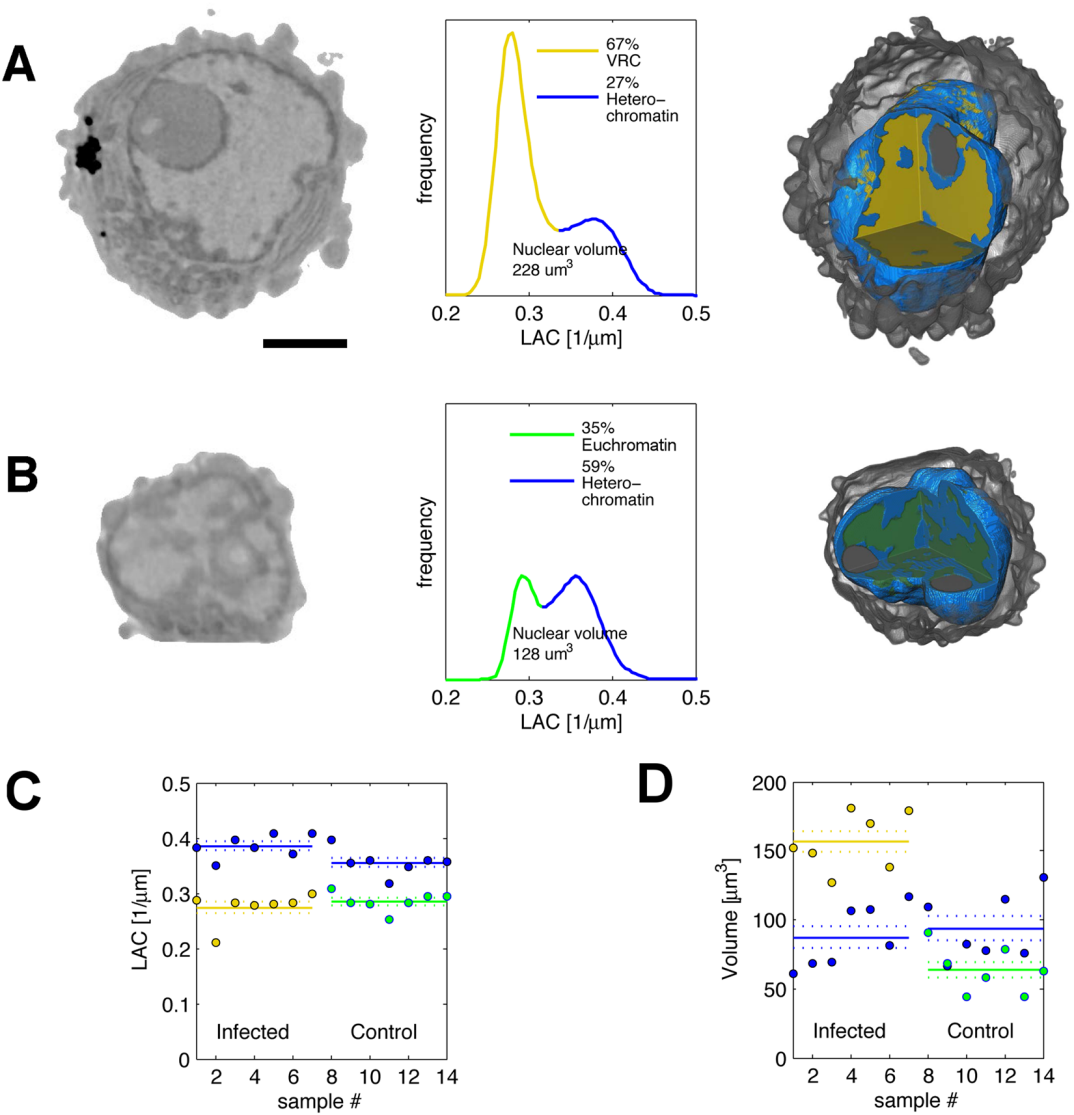
Virus-induced compaction and re-organization of the host chromatin. Nuclear volume of infected cells (**A**) was increased compared with non-infected cells (**B**) to accommodate VRC, as seen in SXT orthoslices (left) LAC-histograms plotting number of voxels with specific LAC values (centre), and 3D views of segmented and color-coded nuclei (right). Scale bar, 3 µm. (**C**) Nuclear LAC values and (**D**) volumes of heterochromatin (blue) and VRC (yellow) of infected cells (n = 7) and volumes of heterochromatin (blue) and euchromatin (green) of non-infected cells. Solid lines represent the mean and dotted lines the mean ± SEM. See also Supplementary Movie S1.

The distinct LAC values of SXT were used to automatically segment nuclear structures in the HSV-1 infected cells. In both the infected and non-infected cells, two populations of differing densities were evident (Fig. 3A and B, Supplementary Movie S1). In the following sections, the high-density population is referred to as heterochromatin and the low-density population either as euchromatin in the non-infected cells or VRC in the infected cells. Because of the similar LAC values of euchromatin and VRC, they cannot be distinguished in the infected cells, but due to the infection-induced marginalization of chromatin, which was also seen in our fluorescence microscopy data, euchromatin is most likely relocated towards the same perinuclear areas where heterochromatin is located (Fig. 1B).

The infection-induced marginalization of the host chromatin had a clear effect on the heterochromatin density, increasing the LAC values of heterochromatin in comparison with those of the non-infected cells (Fig. 3A and B). In the infected cells, the average LAC of heterochromatin was 0.386 ± 0.007 μm^−1^ (n = 7), whereas in the non-infected cells it was 0.356 ± 0.008 μm^−1^ (n = 7) (Fig. 3C). In the infected cells, the highest LAC value was 0.459 ± 0.012 μm^−1^, while it was 0.416 ± 0.009 μm^−1^ in the non-infected cells. This indicated that heterochromatin was more tightly packed in the infected cells. The average LAC value of the VRC, 0.27 ± 0.01 µm^−1^, was relatively similar to (or slightly lower than) that of the euchromatin (*i.e*., in the non-infected cells), 0.285 ± 0.006 μm^−1^ (Fig. 3C). The accuracy of the LAC measurements had been determined earlier by comparing increasing concentrations of BSA and haemoglobin *in vitro* with theoretical LAC values^29^ and with those of alcohol-oxidase crystals in yeast cells^22^.

Plotting the number of voxels (volume) for specific LAC values showed that the average volume of both the nucleus (260 ± 20 μm^3^) and cytoplasm (410 ± 30 μm^3^) were increased in the infected cells (in the non-infected cells they were 170 ± 14 μm^3^ and 178 ± 14 μm^3^, respectively). The surface area of the nucleus also was increased, from 176 ± 9 μm^2^ in the non-infected cells to 260 ± 20 μm^2^ in the infected cells. Most of the increase in nuclear volume was due to the space occupied by the VRC (157 ± 7 μm^3^) – more than twice the volume occupied by euchromatin (64 ± 6 μm^3^, Fig. 3D). In contrast, the average heterochromatin volume remained relatively unaltered as a result of infection (87 ± 8 μm^3^ in infected cells, 94 ± 8 μm^3^ in non-infected cells).

**Figure 4:**
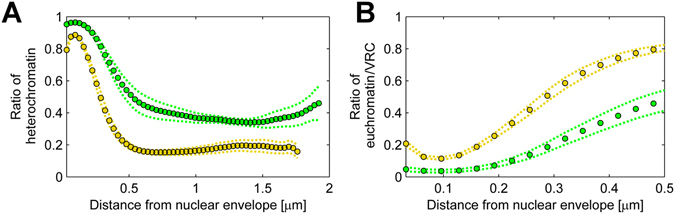
Relative distribution of cellular heterochromatin and viral VRC. (**A**) The relative amount of heterochromatin in infected (yellow) and non-infected (green) cells as a function of distance from the NE. (**B**) The relative amount of euchromatin/VRC in infected (yellow) and non-infected (green) cells at increasing distances from the NE. Dotted error lines represent the mean ± SEM.

To determine the effects of viral infection on the 3D architecture of heterochromatin, we mapped the relative distribution of heterochromatin and euchromatin/VRC as a function of the distance from the NE. This analysis indicated that in the infected cells, 78% of the heterochromatin was concentrated in a band less than 0.5 µm thick in the nuclear periphery, next to the NE (Fig. 4A). This finding is consistent with the immunofluorescence analysis of infected cells, which showed that the heterochromatin marker was concentrated in the nuclear periphery (Fig. 2A). In the non-infected cells, 68% of the heterochromatin was located within 0.5 µm from the NE, and the rest was distributed through the inner nuclear regions (Fig. 4A), with the majority of the low LAC euchromatin. Notably, our analysis indicated that, in the infected cells, 15.0 ± 0.7% of the euchromatin/VRC was located within 160 nm from the NE, in contrast with only 4.1 ± 1.0% in the non-infected cells (Fig. 4B). This shows that infection increased the presence of low LAC nucleoplasm close to the NE, suggesting that the heterochromatin layer is discontinuous along the nuclear rim. In summary, our results indicate that the formation of VRC is accompanied by a profound modification of the nuclear architecture, including the expansion of the nucleus, the marginalization of host heterochromatin, and the emergence of the low-density areas in the nuclear periphery.

### Simulations of the nuclear transport of HSV-1 capsids

To study the intranuclear transport of HSV-1 capsids at late infection, capsid motion was simulated with the time-domain random walk method where the diffusing particles are moved on a lattice and the waiting time between consecutive jumps is an exponentially distributed random variable. The model was used to simulate paths of HSV-1–sized particles (125 nm) in 3D reconstructions of nuclei generated from SXT orthoslices. Our aim was to determine how the chromatin geometry of the infected cells affects the diffusive transport of capsids to the NE.

Diffusion coefficients in the nucleus were set to the value 2 × 10^−2^ µm^2^/s, consistent with the values observed in a recent study^30^. The value was treated as a rough approximation, sufficient for analysing the timescales and properties of transport. Even though the diffusion coefficient was rather low, the diffusion coefficient of chromatin itself in non-infected cells is about one to two orders of magnitude lower than the value used for capsids here^31^. To avoid increased complexity, and to take into account that the infection-induced compaction is likely accompanied by stabilization of chromatin, chromatin motion was not included into the model.

The TEM data and fluorescence data of viral capsid protein VP5, imaged together with DAPI, showed that the viral capsids and VP5 stain were mostly located in the lower-density areas of the nucleus (VRC and euchromatin) but rarely in the denser heterochromatin regions (Fig. 1). Accordingly, the capsid motion was restricted to areas where the LAC values were below 0.34 μm^−1^ (Fig. 3A).

**Figure 5:**
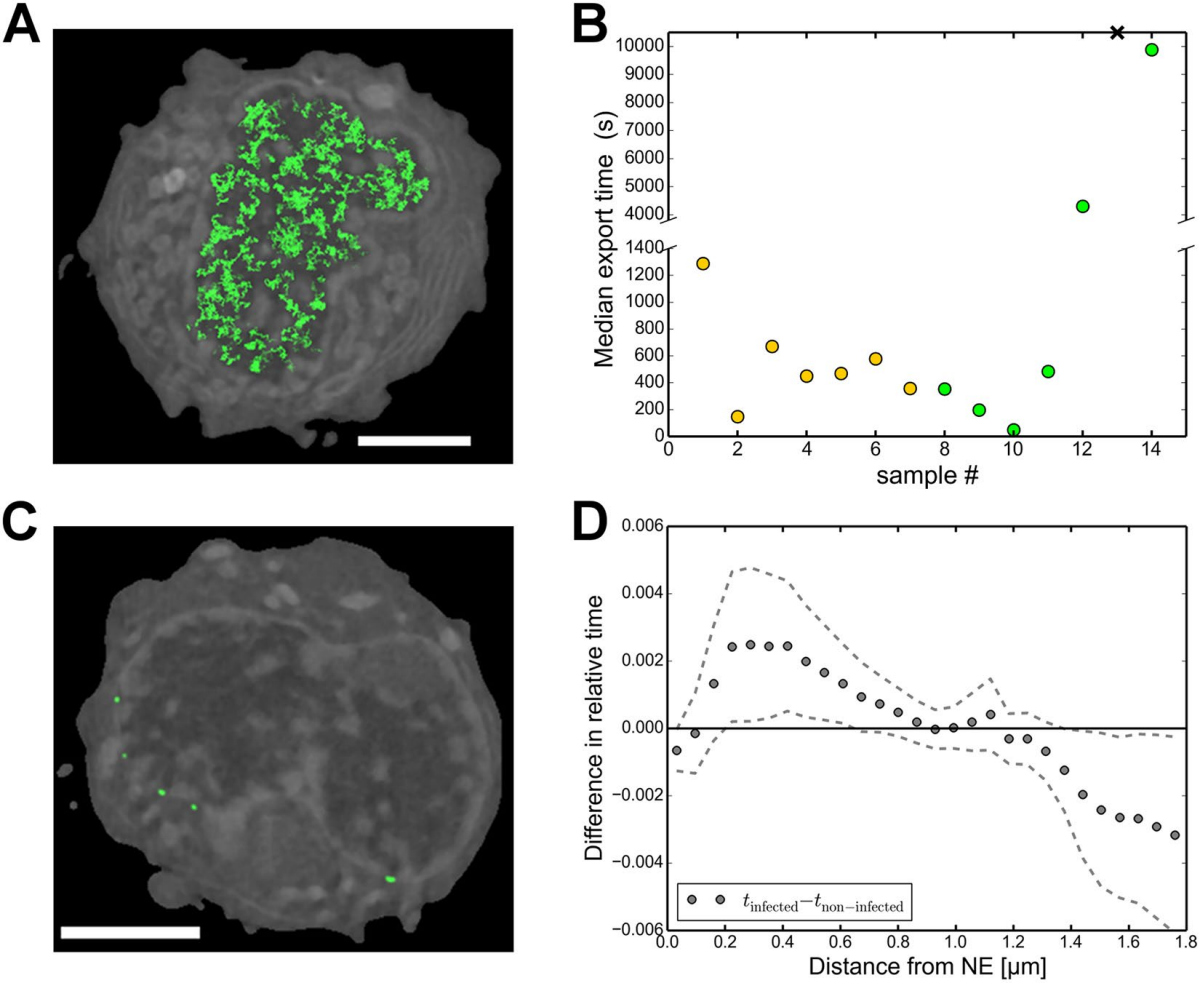
Nuclear transport of capsids to the NE. (**A**) Paths traced by 1000 capsids for 5 seconds (green) near one plane of the simulation geometry. The displayed path has a lower intensity when it is farther away from the SXT orthoslice shown as background (grey). Low and high LAC values of chromatin are indicated with dark and light greyscale values. Scale bar, 3 μm. See also Supplementary Movie S2. (**B**) Median transport times of capsids to the NE for infected (yellow) and non-infected (green) cells. The control cell with a cross symbol did not allow passage of capsids, and the median time is therefore not defined for it. The break in the *y*-axis separates low and high transport times. (**C**) Low-density nuclear egress sites of capsids (green) in an SXT cross-section of an infected cell. Scale bar, 3 μm. See also Supplementary Movie S3. (**D**) Difference in the relative amount of time spent by capsids at various distances from the NE between the infected and non-infected cells. Dotted error lines represent the mean ± SEM.

First, we simulated the motion of 30,000 virus-sized particles in every cell (n = 7, Fig. 5A and Supplementary Movie S2). The simulations showed that in the infected cell nuclei the median time for a randomly placed particle to reach the NE ranged between 2.5 and 21 minutes (Fig. 5B), while the average of these values over the cells was 9 ± 2 minutes (average ± SEM). Since almost all the capsids (99.9%) were eventually (within the total simulated time of four hours) able to reach the NE, the nuclear chromatin had few areas enclosing and trapping the capsids inside. Next, we studied the restrictive effect caused by heterochromatin to transport times by allowing the capsids to move freely in the entire nucleus, regardless of the chromatin density. In these simulations, the median time to reach the NE was only about 15 seconds, which demonstrates that capsid transport to the NE is significantly limited by the presence of peripheral chromatin.

To study how the infection-induced changes in the chromatin geometry and density affect the capsid transport we also simulated capsid diffusion in the non-infected control cell nuclei. These studies revealed that transport times to the NE varied significantly. In four cells, the median time to reach the NE was relatively similar to that of the infected cells, but in the other three cells, the transport was either very slow or did not occur at all (Fig. 5B). This suggests that the chromatin distribution varied strongly between the non-infected cells. Some of the cells contained pathways through the chromatin that were wide enough to allow the simulated passage of capsids, whereas in others the chromatin layer did not allow capsid transport.

Our earlier studies disclosed the induction of low-density virus-sized channels across the host chromatin in infection^7^. To examine the presence of export locations at the NE, we recorded the positions where the capsids arrived at the NE during the simulations (Fig. 5C and Supplementary Movie S3). In the infected cells on average 1.9 ± 0.5% of the lattice sites defining the nuclear envelope had a capsid arriving to them during the simulation. In the non-infected cells the value was 2.1 ± 0.8%. In the infected cells, there were 150 ± 20 separate export locations at the NE, whereas in the non-infected cells there were 37 ± 17 export locations.

Finally, to study the effect of the marginalized chromatin on the distribution pattern of capsids over time, we recorded the total time spent by the particles for every lattice point in the nucleus. The lattice sites were then grouped based on their distance from the nuclear envelope, and the relative amount of time that the particles spent at every distance was calculated. Our analysis revealed that in the infected cells the capsids spent relatively longer time near the nuclear periphery than in the non-infected cells (Fig. 5D). A possible explanation for this behaviour is that in the control cells the capsids at these locations were already inside the peripheral chromatin layer, while in the infected cells, due to the chromatin being displaced more towards the edge, the capsids were still outside the chromatin layer, trying to find paths through it (Fig. 5D). This finding is consistent with our SXT analyses (Fig. 3), which showed that heterochromatin was located closer to the NE in infected cells than in non-infected cells. In the non-infected cells the capsids spent more time in the central parts of the nucleus, which can be explained by the more central distribution of chromatin which restricted the motion away from there. We also observed that a border within 0.5 μm from the NE restricted the flux of particles beyond this region (Supplementary Figure S4). This further shows that peripheral chromatin causes capsids to spend more time in the central parts of the nucleus devoid of chromatin, possibly diffusing around and seeking routes through the chromatin layer around them.

In summary, our simulations using the random walk model in 3D SXT reconstruction verified that the diffusion of viral capsids is limited by the compacted peripheral chromatin and that low-density channels through the chromatin layer are required to facilitate the viral transport and nuclear egress.

## Discussion

Although HSV-1 infection is known to cause marginalization of the host chromatin, the quantitative effects on the density and organization of chromatin, and whether chromatin limits capsid translocation towards the NE, are not known. By combining SXT, TEM and confocal imaging techniques with advanced image analysis and numerical modelling, we revealed the condensation of chromatin and restricted mobility of the viral capsids in it.

Our studies confirmed that the emergence of an enlarged VRC was accompanied by the exclusion of host chromatin and its marginalization into the nuclear periphery. The analysis of heterochromatin distribution showed that it was enriched in the nuclear periphery both in infected and non-infected cells. Because the heterochromatin distribution did not change much during the infection, it is probable that the observed exclusion of chromatin from the nuclear centre is due to active euchromatin being relocated towards the nuclear periphery of the infected cells. This is in line with earlier HSV-1 studies showing that the expression of some genes is maintained or increased in infection^10–13^. Moreover, HSV-1 proteins are known to be able to influence chromatin status towards either the loosely packed, transcriptionally active euchromatin, or towards inactive condensed heterochromatin^14–16, 24, 25, 32–34^.

Our SXT analysis showed that the late stage of HSV-1 infection triggers an extensive modification of nuclear size and host chromatin organization. Both the mean volume of the nucleus and cytoplasm were increased in the infected cells. Notably, heterochromatin enrichment near the nuclear periphery was accompanied by compaction of the host chromatin. The HSV-1-induced changes in the nucleus have been studied intensely by light- and electron-based imaging techniques. These studies have verified increases in the nuclear volume and marginalization of the host genome^5, 6, 35^. However, these approaches are confronted with fundamental limitations in 3D imaging of the architecture of the entire nucleus and/or in quantitative analysis of the molecular composition of nuclear chromatin compartments. SXT imaging circumvents these shortcomings by using comparatively short wavelengths of soft x-rays, which enables high-resolution 3D imaging of the nuclear architecture and quantitative assessment of the molecular density of nuclear structures including chromatin^7, 22, 36^.

Our simulations of particle distribution in a 3D SXT chromatin reconstruction revealed that the marginalized chromatin indeed acts as an obstacle to the virus-sized particle motion. This suggests that the compact layer of host heterochromatin constitutes an accessibility barrier for the translocation of viral capsids towards the inner nuclear membrane across which they exit the nucleus. However, infection-induced breaks in the marginalized chromatin enable the capsid motion through it^7^. The simulations showed that the virus-sized particles were able to move to numerous locations at the NE in the infected cell chromatin geometries. This suggests that at any given moment there are sufficient channels through the chromatin to allow the passage of capsids. Moreover, our results demonstrate that the immobile chromatin network assumed in the simulations is sufficient for capsid egress, suggesting that chromatin motion is not necessarily required for it. Furthermore, diffusion seems to be an adequate method for capsid transport to the NE, which supports the idea of passive transport of capsids in the nucleus. This is in line with recent studies concluding that the motility of capsids is a process based on passive diffusion^30, 37^.

Studying the capsid motion *in silico* allows us to change the details of the model and to examine a hypothetical case where the capsids can move regardless of the surrounding chromatin densities. When the capsids were allowed to travel in the entire nucleus, without the presence of restricting chromatin, capsid transport to the NE was significantly faster. This reveals the strong effect that the exclusion of capsids by the dense chromatin has on capsid export dynamics. Comparing the transport in infected cells to those in control cells, we observed that the transport times to the NE were more consistent in the infected cells than in the non-infected cells. This suggests that the changes to the chromatin geometry, low-density channels across the host chromatin in particular, are beneficial for capsid egress.

In summary, SXT imaging combined with advanced data analysis allowed us, for the first time, to demonstrate the HSV-1-induced molecular compaction of host chromatin. The modelling in the 3D SXT reconstruction of chromatin demonstrated that condensed chromatin in the infected cell nucleus constitutes an accessibility barrier and that there exist interchromatin channels allowing capsid transport to multiple locations at the NE.

## Materials and Methods

### Cells and viruses

To allow the long-term growth of B lymphocytes, the Abelson murine virus was used to infect them and to produce immortalized pre-B cell lines of a female mouse^38^. The original cells were a gift from Barbara Panning (UCSF School of Medicine, Biochemistry and Biophysics, San Francisco, CA, USA). Cells were maintained as suspension cultures by a procedure described in ref. 7. The HSV-1 strain 17+ expressing EYFP-ICP4 (vEYFP-ICP4) was a generous gift from R. Everett (MRC Virology Unit, Glasgow, Scotland, UK^39^). The viruses had been isolated as described in ref. 39. To infect the cells, they were inoculated with HSV-1 or HSV-1 EYFP-ICP4 at an MOI of 5–10 and kept at 37 °C until live-cell microscopy, fixation, and SXT analysis.

### Confocal microscopy studies

For immunolabelling studies, cells were infected with HSV-1 or vEYFP-ICP4 HSV-1 at an MOI of 5. The cells were collected by centrifugation (400 RCF for 5 min) at 24 h p.i., spread and air dried on Zeiss high performance cover glasses (D = 0.17 mm, size 18 × 18 mm), after which they were fixed with 4% paraformaldehyde (PFA; 20 min at room temperature [RT]). Capsid protein VP5-specific monoclonal antibody (MAb; Santa Cruz Biotechnology Inc. Dallas, TX, USA) was used to detect viral capsids, followed by goat anti-mouse Alexa 594 secondary antibody (Ab; Thermo Fisher Scientific, Massachusetts, USA). Modified histones were labelled with rabbit antibody against H3K9me3 (Abcam, Cambridge, UK) followed by goat anti-rabbit Atto 647 (Abcam) conjugated secondary Ab. The nuclear lamina was detected with a lamin B1-specific Ab (Abcam) followed by anti-rabbit Alexa 647 conjugated secondary Ab (Thermo Fisher Scientific). DNA was stained in the embedding stage with 4′-6-diamidino-2-phenylindole (DAPI)-containing ProLong antifade reagent (Thermo Fisher Scientific).

The immunolabelled cells were imaged using Nikon A1R laser scanning confocal microscope (Nikon Instruments Inc., Melville, USA) with CFI Plan Apo VC 60XH oil immersion objective (N.A 1.4). DAPI and EYFP were excited with a 405 nm diode laser and a 514 nm argon laser, respectively. DAPI fluorescence was detected with a 450/50 nm band-pass filter and EYFP fluorescence with a 540/30 nm band-pass filter. A 561 nm sapphire laser was used to excite Alexa 594, and the fluorescence was collected with a 595/50 nm band-pass filter. Atto 647 and Alexa 647 were excited with a 642 nm diode laser and the fluorescence was collected with a 660 nm long-pass filter. Stacks of 512 × 512 pixels were collected with a pixel size of 50 nm/pixel in the x- and y- directions, and 150 nm in the z-direction. Images were iteratively deconvoluted with Huygens Essential software (SVI, the Netherlands) using a signal-to-noise ratio of 7 and quality threshold of 0.0l. Image analysis was done with ImageJ.

Co-localization analyses were done in ImageJ software using the JACoP plugin^40^ with manually adjusted threshold values. The images were first denoised with a Gaussian blur, sigma = 2, and then a binary image was generated with the Otsu threshold. After filling any holes, the amount and volume of objects were determined using the 3D Objects Counter plugin^40^. Distance analysis of the fixed cell samples was done by first making a Euclidian distance map from an image of interest using Exact Euclidian Distance Transform (3D) plugin in ImageJ, and then comparing the original image against the distance map using an in-house Java code. The distance values were sorted to 0.1 μm-wide bins and the average intensity of the studied label was calculated for each bin.

### Transmission electron microscopy

Infected cells and non-infected control cells were fixed in 4% paraformaldehyde and 0.25% glutaraldehyde in 50 mM phosphate buffer (at a pH of 6.8) followed by post-fixation in 1% OsO4 for 1 h on ice. Cells were dehydrated with ethanol and then embedded in low-viscosity embedding resin (TAAB Laboratories Equipment Ltd, UK). Thin sections were cut by Ultracut UC6a ultramicrotome (Leica Mikrosysteme GmbH, Germany) followed by collection on Pioloform-coated, single-slot copper grids. Sections were double stained with 2% aqueous uranyl acetate and lead citrate and examined using TEM JEOL JEM1400 (JEOL Ltd., Tokyo, Japan), operated at 80 kV. The images were recorded using a bottom-mounted Quemesa CCD camera with 4008 × 2664 pixel resolution (EMSIS GmbH, Münster Germany).

### Soft x-ray tomography

By a procedure described in ref. 7, mouse B cells were infected with vEYFP-ICP4 HSV-1, prepared and frozen into glass capillaries, and imaged using an XM-2 soft x-ray microscope in the National Center for X-ray Tomography (http://ncxt.lbl.gov) located at the Advanced Light Source (http://www.als.lbl.gov) of Lawrence Berkeley National Laboratory. Capillaries were kept in a stream of liquid nitrogen-cooled helium gas during data collection^41, 42^. This allowed data collection without any observable radiation damage. Each dataset contained 90–180 projection images collected sequentially around a rotation axis in 1–2° increments, which gives a total rotation of 180°, using a 300–400 ms exposure time. The voxel size was 32 nm. Projection images were normalized^26^ and then manually aligned using the IMOD software. Finally, tomographic reconstructions were calculated using iterative reconstruction methods^43, 44^. LAC values were determined as described previously^45^.

### Numerical simulations of capsid motion

Capsid diffusion in nuclei was simulated using the time-domain random walk method^46–49^, where the probability for a random walker to jump from one lattice site to another is proportional to the harmonic average of the diffusion coefficients of the two sites and the time interval between two jumps is an exponentially distributed random variable. The simulation geometry was constructed using the SXT 3D images of the cells and, to get a more refined discrete representation of the diffusion dynamics, the lattice defined by the 32 nm pixels of the SXT images was subdivided by a factor of 4 to yield a lattice spacing of 8 nm for the simulations. The nuclei were divided into allowed regions, where the particle could diffuse freely, and into forbidden regions, where the particle was not allowed to travel, by setting a chromatin density limit for the particle motion. This density limit was defined as the minimum point between the two density populations in the LAC histogram (Fig. 3). The forbidden regions consisted of those areas where the centre of the particle was closer than its radius (62.5 nm) to a region that had too high a chromatin density. In the beginning of the simulation particles were placed randomly in the allowed regions of the nucleus, and during the simulations the arrival times of the particles to the NE and the arrival locations were recorded for each particle. From the arrival locations of the particles at the NE, distinct exit locations were identified by grouping contiguous pixels together. The number of particles was 30000 for every simulation.

## Electronic supplementary meterial

Supplementary Information

Supplementary Movie 1

Supplementary Movie 2

Supplementary Movie 3
