# Supplementary material for: Chromatin organization regulates viral egress dynamics
Source: Sci Rep. 2017 Jun 16;7:3692. doi: 10.1038/s41598-017-03630-y (PMC5473834; doi:10.1038/s41598-017-03630-y)
Supplement: Supplementary file 1 — Supplementary Information [file 41598_2017_3630_MOESM1_ESM.pdf]

Chromatin organization regulates viral egress dynamics

Vesa Aho[1], Markko Myllys[1+], Visa Ruokolainen[2+], Satu Hakanen[2], Elina Mäntylä[2], Jori Virtanen[3], Veijo Hukkanen[4], Thomas Kühn[5,6], Jussi Timonen[1,7], Keijo Mattila[1,8], Carolyn A. Larabell[9,10], and Maija Vihinen-Ranta[2*]

[1] Department of Physics, and Nanoscience Center, University of Jyväskylä, Jyväskylä, Finland
[2] Department of Biological and Environmental Science, and Nanoscience Center, University of Jyväskylä, Jyväskylä, Finland
[3] Institute of Biotechnology, University of Helsinki, Helsinki, Finland
[4] Faculty of Medicine, University of Turku, Turku, Finland
[5] Department of Applied Physics, University of Eastern Finland, Kuopio, Finland
[6] Finnish Meteorological Institute, Kuopio, Finland
[7] ITMO University, Saint Petersburg, Russia
[8] Department of Physics, Tampere University of Technology, Tampere, Finland
[9] Department of Anatomy, University of California San Francisco, San Francisco, California, USA
[10] Physical Biosciences Division, Lawrence Berkeley National Laboratory, Berkeley, California, USA
[*]Corresponding author, E-mail: maija.vihinen-ranta@jyu.fi
[+]These authors contributed equally to this work

SUPPLEMENTAL FIGURES

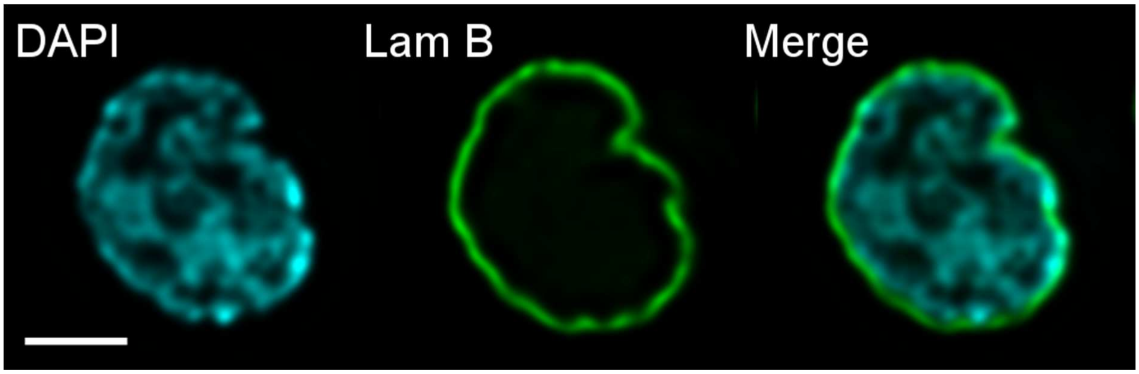

Figure S1. Distribution of chromatin and lamin B in a non-infected cell. Confocal microscopy images of a non-infected cell with DAPI (cyan) and lamin B antibody (green) labelling. Scale bar, 3 µm.

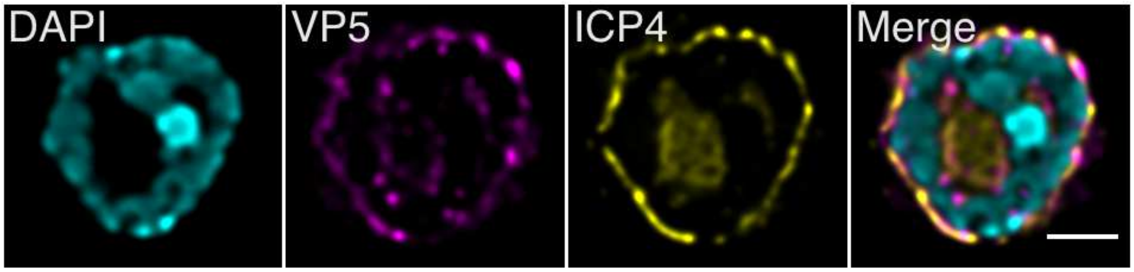

Figure S2. Peripheral distribution of host chromatin and viral proteins. Confocal microscopy images of an EYFP-ICP4 HSV-1 infected cell. Distribution of the viral VP5 (magenta) and EYFP-ICP4 (yellow) proteins together with DAPI-labelled chromatin (cyan) at 24 h p.i. Scale bar, 3 µm.

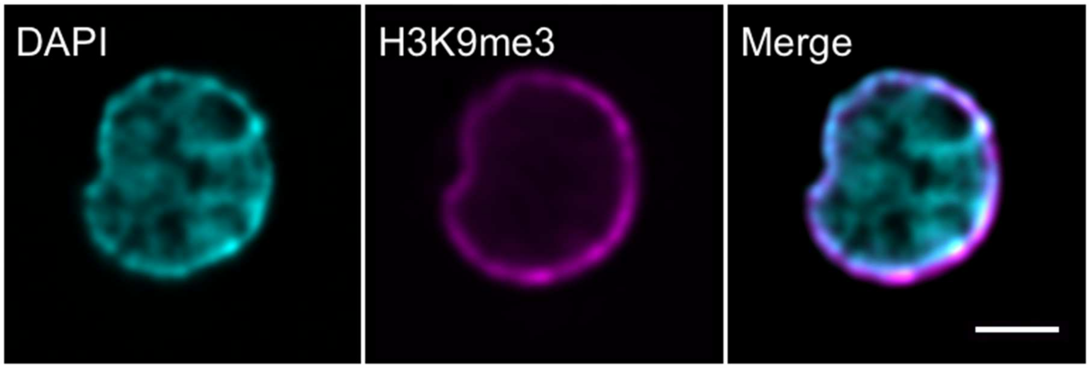

Figure S3. Distribution of heterohromatin marker in a non-infected cell. Immunofluorescence analysis of the distribution of heterochromatin marker H3K9me3 (magenta) and DAPI (cyan). Scale bar, 3 µm.

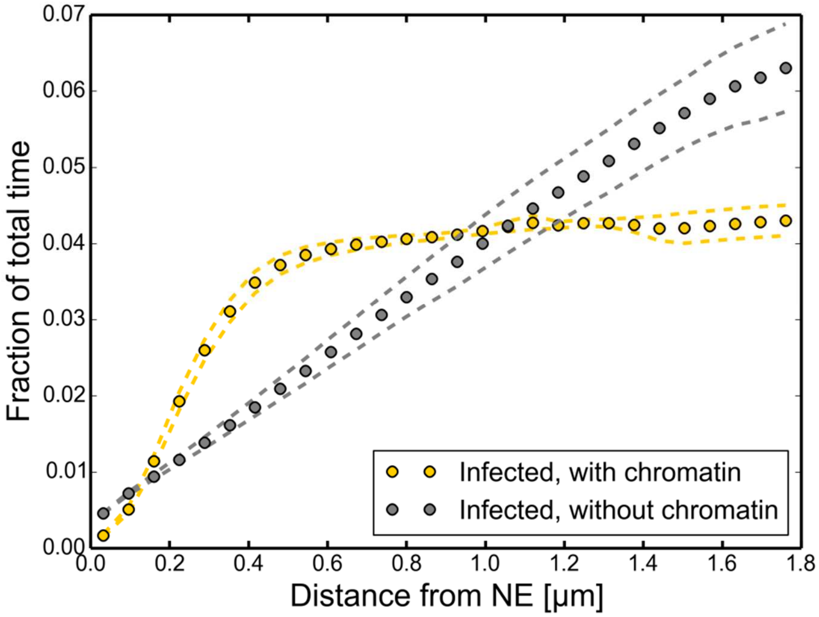

Figure S4. Time spent by the capsids in the nucleus. The relative time spent by the capsids in the infected cell nuclei as a function of distance from the nuclear envelope with the restricting effect of chromatin (yellow) and without it (dark grey). The relative time spent decreases when moving closer to the nuclear envelope due to outward flux of particles at the nuclear envelope (particles are removed from the simulation once they reach the nuclear envelope).

SUPPLEMENTAL MOVIES

Movie S1. 3D SXT presentation of nuclear organization. A 3D reconstruction of SXT orthoslices of an infected (upper left) and a non-infected (upper right) cell. Segmented and colour-code selected nuclear structures of the infected (lower left) and the non-infected (lower right) cell based on SXT LAC values. The colours show heterochromatin (blue), VRC (yellow), and euchromatin (green). Scale bars, 1 and 10 µm.

Movie S2. Simulated capsid paths. Paths traced in real time (during 10 seconds) by 1000 capsids (green) near one plane of the simulation geometry. The displayed path has a lower intensity when located farther away from the SXT orthoslice shown as background (grey). Low and high LAC values of chromatin are indicated with dark and light greyscale values.

Movie S3. Simulated export locations. Animation of SXT orthoslices of the x-ray tomographic reconstruction of an infected cell (grey), showing the locations where capsids have reached the NE in the simulation (green). Notice the low-density gaps through the peripheral chromatin at the export regions. Low and high LAC values of chromatin are indicated with dark and light greyscale values.
